# Laryngeal NUT Carcinoma With *BRD4* :: *NUTM1* Fusion: A 4‐Year Clinical Course Highlighting the Limitations of Current Multimodal Therapy

**DOI:** 10.1002/cnr2.70679

**Published:** 2026-09-08

**Authors:** Masafumi Kanno, Chiai Sasaki, Eiichi Kato, Hoshino Terada, Nobuhiro Hanai, Eiichi Sasaki, Mana Fukushima, Toshiki Masuishi, Keisuke Takanari, Yasuto Fukada, Yuto Miyazaki, Yuki Sonoda, Yukinori Kato, Taiyo Morikawa, Tetsuji Takabayashi, Shigeharu Fujieda

**Affiliations:** ^1^ Division of Otorhinolaryngology—Head and Neck Surgery, Department of Sensory and Locomotor Medicine, Faculty of Medical Sciences University of Fukui Fukui Japan; ^2^ Department of Head and Neck Surgery Aichi Cancer Center Hospital Nagoya Japan; ^3^ Department of Pathology and Molecular Diagnostics Aichi Cancer Center Hospital Nagoya Japan; ^4^ Department of Pathology, Faculty of Medical Sciences University of Fukui Fukui Japan; ^5^ Department of Clinical Oncology Aichi Cancer Center Hospital Nagoya Japan; ^6^ Department of Plastic and Reconstructive Surgery Aichi Cancer Center Hospital Nagoya Japan; ^7^ Department of Plastic and Reconstructive Surgery Okayama University Okayama Japan

**Keywords:** BET inhibitor, *BRD4*::*NUTM1* fusion, epigenetic therapy, laryngeal NUT carcinoma, VDC/IE chemotherapy

## Abstract

**Background:**

Nuclear protein in testis (NUT) carcinoma is a rare, aggressive malignancy driven by NUTM1 rearrangement, often misdiagnosed as squamous cell carcinoma, with no established standard therapy. This report describes the clinical course and treatment resistance in primary laryngeal NUT carcinoma and provides insights into potential mechanism‐based therapeutic approaches.

**Case:**

A 30‐year‐old man with a laryngeal tumor was initially treated for poorly differentiated squamous cell carcinoma. Recurrent disease prompted re‐evaluation; NUT carcinoma was diagnosed by NUT immunohistochemistry, and the *BRD4*::*NUTM1* fusion was confirmed by molecular analysis. A bromodomain and extra‐terminal (BET) inhibitor and alternating vincristine, doxorubicin, and cyclophosphamide (VDC)/ifosfamide and etoposide (IE) chemotherapy induced transient tumor regression, whereas vorinostat, immune checkpoint inhibitors, and other systemic therapies were ineffective. Disease progression was ultimately driven by uncontrolled locoregional recurrence. Autopsy revealed a massive infiltrative tumor with extensive locoregional invasion, whereas distant metastatic disease was limited to minute pulmonary nodules; death resulted from overwhelming infection secondary to uncontrolled locoregional progression rather than systemic metastasis.

**Conclusion:**

Early molecular diagnosis and biologically informed multimodal strategies are essential for managing laryngeal NUT carcinoma and overcoming treatment resistance.

## 1 Introduction

Nuclear protein in testis (NUT) carcinoma is an epithelial malignancy defined by oncogenic rearrangements of the *NUTM1* gene [1]. Most cases involve fusion of *NUTM1* with *BRD4*, whereas *BRD3* and *NSD3* constitute smaller but established fusion partners [2, 3, 4, 5, 6]. These fusion events alter chromatin regulation and enforce an arrest in cellular differentiation, driving the rapid clinical progression characteristic of this disease [7]. Mechanistically, the fusion protein retains the tandem bromodomains of BRD4, which bind acetylated histones, whereas the NUT moiety recruits p300/CBP, thereby generating large hyperacetylated chromatin domains that drive MYC expression and block squamous differentiation. Since the first report of a t(15;19)‐positive thymic carcinoma in 1991 [8], NUT carcinoma has been recognized across a broad age spectrum, with a median age of 24–26 years [6, 9, 10]. Although traditionally regarded as a midline tumor arising from the thorax or head and neck, *NUTM1*‐rearranged neoplasms have also been identified in non‐midline tissues, such as the salivary glands and thyroid [11, 12, 13, 14, 15].

Diagnosis can be difficult because the histological appearance overlaps substantially with poorly differentiated squamous cell carcinoma (SCC) or other high‐grade small round‐cell tumors [16, 17]. Routine histopathological and immunohistochemical findings are often insufficient to distinguish these tumors from conventional SCC, and confirmation generally requires demonstration of diffuse nuclear NUT immunoreactivity and/or detection of a *NUTM1* rearrangement by FISH or molecular testing [12, 18]. Misclassification at initial presentation is well documented; several tumors were originally diagnosed as Ewing sarcoma or primitive neuroectodermal tumor before molecular confirmation established a definitive diagnosis of NUT carcinoma [19, 20]. These diagnostic limitations underscore the importance of considering NUT carcinoma when encountering an aggressive tumor in a young, non‐smoking patient lacking typical risk factors.

Laryngeal NUT carcinoma is exceptionally rare, with fewer than 20 cases reported to date. Reported patients have ranged from children to older adults, indicating that this disease can occur across a broad age spectrum [21, 22]. Outcomes remain poor despite multimodal treatment with surgery, radiotherapy, and systemic therapy; only isolated reports have explored the use of immune checkpoint inhibitors or epigenetic‐targeted approaches [16, 17].

Herein, we report a rare adult case of laryngeal NUT carcinoma with a prolonged clinical course of approximately 4 years from initial presentation to death, illustrating the diagnostic challenges and therapeutic limitations of this disease.

## 2 Case

### 2.1 Initial Presentation and Primary Treatment (20X0)

**FIGURE 1:**
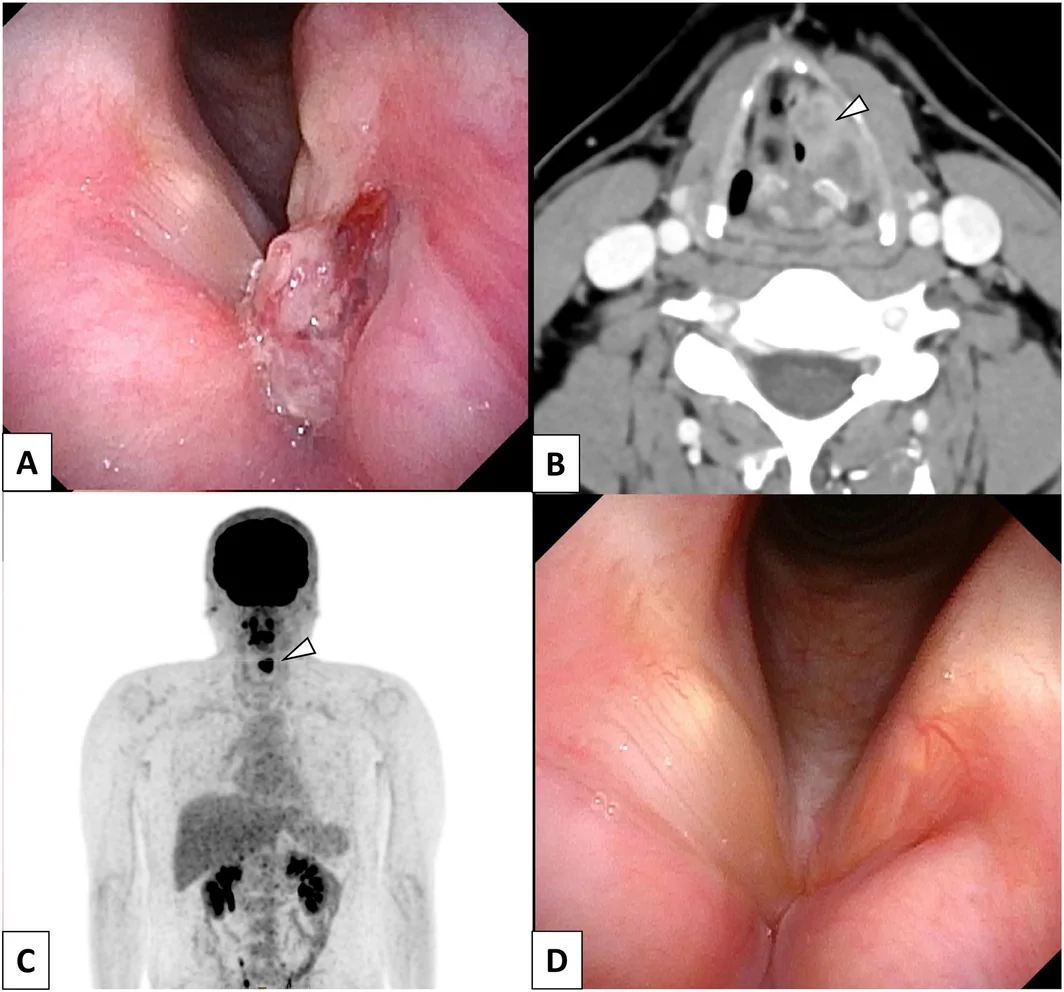
Endoscopic and imaging findings at initial presentation and after definitive therapy. (A) Flexible laryngoscopy demonstrating a tumor extending from the anterior commissure to the left true vocal fold. (B) Contrast‐enhanced CT showing a mass involving the left true vocal fold (white arrow) without evidence of thyroid cartilage invasion. (C) PET/CT demonstrating increased fluorodeoxyglucose (FDG) uptake confined to the primary laryngeal lesion (white arrow), with no evidence of nodal or distant metastasis. (D) Flexible laryngoscopy after completion of definitive chemoradiotherapy showing complete resolution of the primary lesion. CT, computed tomography; PET, positron emission tomography.

A 30‐year‐old man with no history of smoking or alcohol use presented with persistent hoarseness following an upper respiratory infection in May 20X0. Flexible nasolaryngoscopy revealed a tumor arising from the left true vocal fold and extending toward the anterior commissure, without evidence of vocal fold paralysis (Figure 1A). Contrast‐enhanced computed tomography (CT) demonstrated a mass involving the left glottic and supraglottic regions without cervical lymphadenopathy or thyroid cartilage invasion (Figure 1B).

Histopathological examination of a biopsy specimen obtained under general anesthesia in June 20X0 revealed a poorly differentiated SCC characterized by a high nuclear‐to‐cytoplasmic ratio, focal keratinization, and patchy p16 positivity involving approximately half of the tumor cells; abrupt keratinization was not evident in the initial biopsy specimen. HPV‐specific DNA/RNA testing was not performed, and p16 staining was patchy rather than block‐positive. Because the tumor was interpreted as conventional SCC at this stage, NUT immunohistochemistry was not performed at the initial diagnosis. Positron emission tomography (PET) showed abnormal uptake confined to the primary lesion, with no evidence of regional nodal or distant metastasis (Figure 1C). The disease was clinically staged as cT2N0M0 (Stage II). The patient's detailed clinical course and treatment timeline are summarized in Table 1.

**TABLE 1:** Clinical course and treatment timeline.

Line	Period	Regimen/intervention	Best response	Duration of control
First	20X0.06–07	Induction TPEx (docetaxel, cisplatin, cetuximab)	Progression	Followed by CCRT
	20X0.08–10	Definitive CCRT (70 Gy IMRT + carboplatin/5‐FU)	Complete response	10 months
—	20X0.11–20X1.07	Maintenance therapy (oral S‐1)	Complete response (maintained)	9 months
—	20X1.09	First salvage surgery (total laryngectomy)	Local control	6 months
Second	20X2.03	TP (cisplatin, docetaxel)	Progression	< 1 month
Third	20X2.04	Nivolumab	Progression	< 1 month
—	20X2.05	Second salvage surgery (pharyngectomy, jejunal reconstruction)	Local control	5 months
—	20X2.11	Third salvage surgery (revision surgery)^a^	Local control	2 months
Fourth	20X3.01–07	BET inhibitor (clinical trial)	Marked partial response	6 months
Fifth	20X3.09	Paclitaxel + cetuximab	Progression	< 1 month
Sixth	20X3.11–20X4.03	VDC/IE (alternating cycles)	Marked partial response	4 months
Seventh	20X4.03–04	Pembrolizumab + cisplatin/5‐FU	Progression	< 1 month
Eighth	20X4.05	Vorinostat (HDAC inhibitor)	Progression	< 1 month
Ninth	20X4.06	Paclitaxel + cetuximab (retreatment)	Progression	< 1 month

Abbreviations: BET, bromodomain and extra‐terminal; CCRT, concurrent chemoradiotherapy; HDAC, histone deacetylase; IMRT, intensity‐modulated radiotherapy; TPEx, docetaxel, cisplatin, and cetuximab; VDC/IE, vincristine, doxorubicin, and cyclophosphamide/ifosfamide and etoposide.

^a^ The definitive diagnosis of NUT carcinoma was established by NUT immunohistochemistry performed on the resected specimen.

Given the patient's young age, a larynx‐preserving definitive treatment strategy was selected. Induction chemotherapy with docetaxel, cisplatin, and cetuximab (TPEx) was administered from June to July 20X0, followed by definitive chemoradiotherapy consisting of intensity‐modulated radiation therapy to a total dose of 70 Gy with concurrent carboplatin and 5‐fluorouracil from August to October 20X0. Posttreatment flexible nasolaryngoscopy demonstrated complete resolution of the primary lesion (Figure 1D), which was confirmed by follow‐up CT. Maintenance therapy with oral S‐1 was subsequently initiated.

### 2.2 First Local Recurrence and Initial Salvage Surgery (20X1)

**FIGURE 2:**
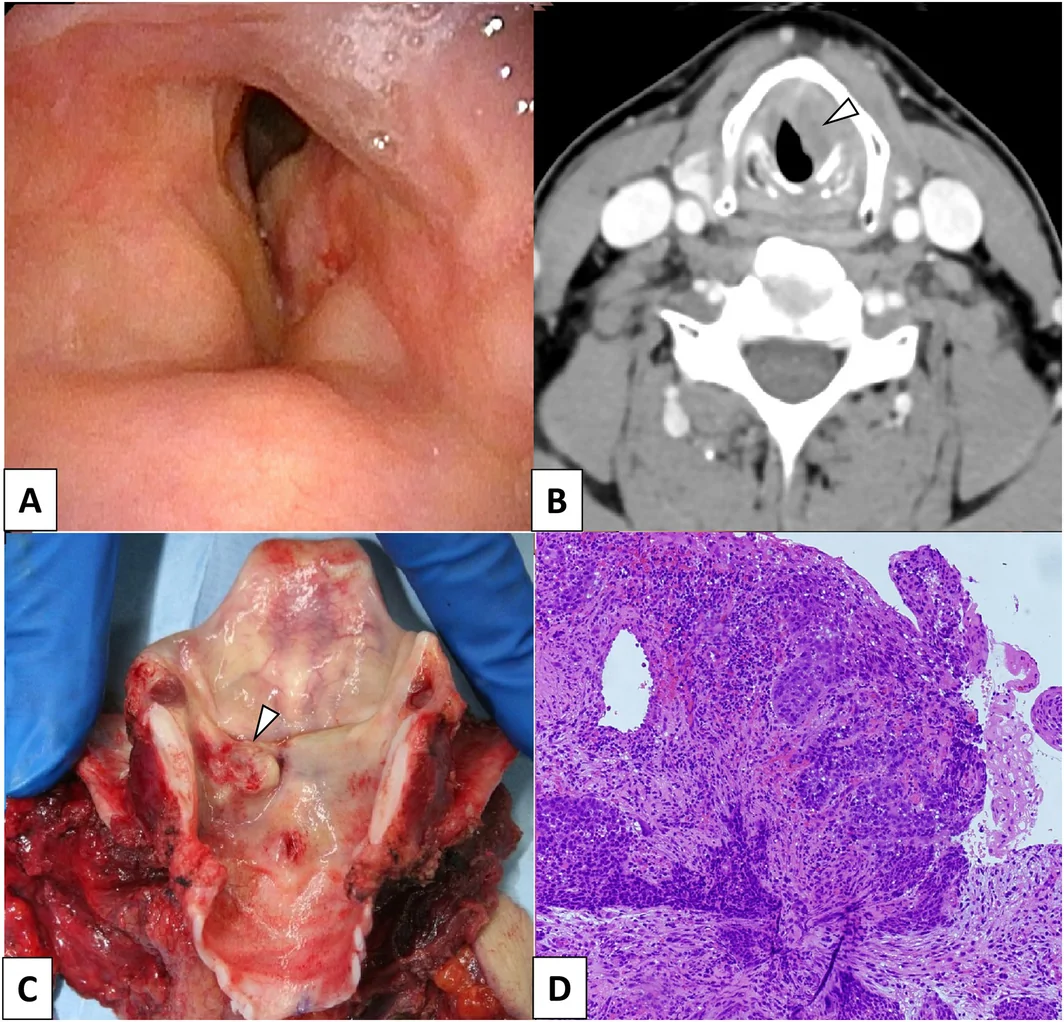
Endoscopic, radiologic, and histopathological findings at the first local recurrence. (A) Laryngoscopic view showing a recurrent tumor of the left vocal fold with associated ipsilateral vocal fold paralysis. (B) Contrast‐enhanced CT image demonstrating a recurrent mass confined to the left supraglottic region (white arrow). No significant cervical lymphadenopathy is observed. (C) Macroscopic finding from the specimen obtained from salvage total laryngectomy. The tumor extends into the laryngeal ventricle (white arrow). (D) Histopathological image of the resected specimen (hematoxylin and eosin stain, original magnification ×100), consistent with moderately to poorly differentiated squamous cell carcinoma. CT, computed tomography.

After completion of chemoradiotherapy, close surveillance with monthly examinations was undertaken. Ten months later, in August 20X1, flexible nasolaryngoscopy revealed a recurrent lesion at the primary site associated with new‐onset ipsilateral vocal fold paralysis (Figure 2A). Imaging demonstrated a recurrent mass confined to the left supraglottic region without cervical lymphadenopathy (Figure 2B).

In September 20X1, salvage total laryngectomy was performed, achieving complete resection of a tumor extending into the laryngeal ventricle (Figure 2C). Histopathological examination demonstrated a moderately to poorly differentiated SCC, consistent with recurrent disease (Figure 2D). The postoperative course was uneventful, and no adjuvant therapy was administered.

### 2.3 Second Recurrence: Posterior Pharyngeal Wall Lesion (20X2)

**FIGURE 3:**
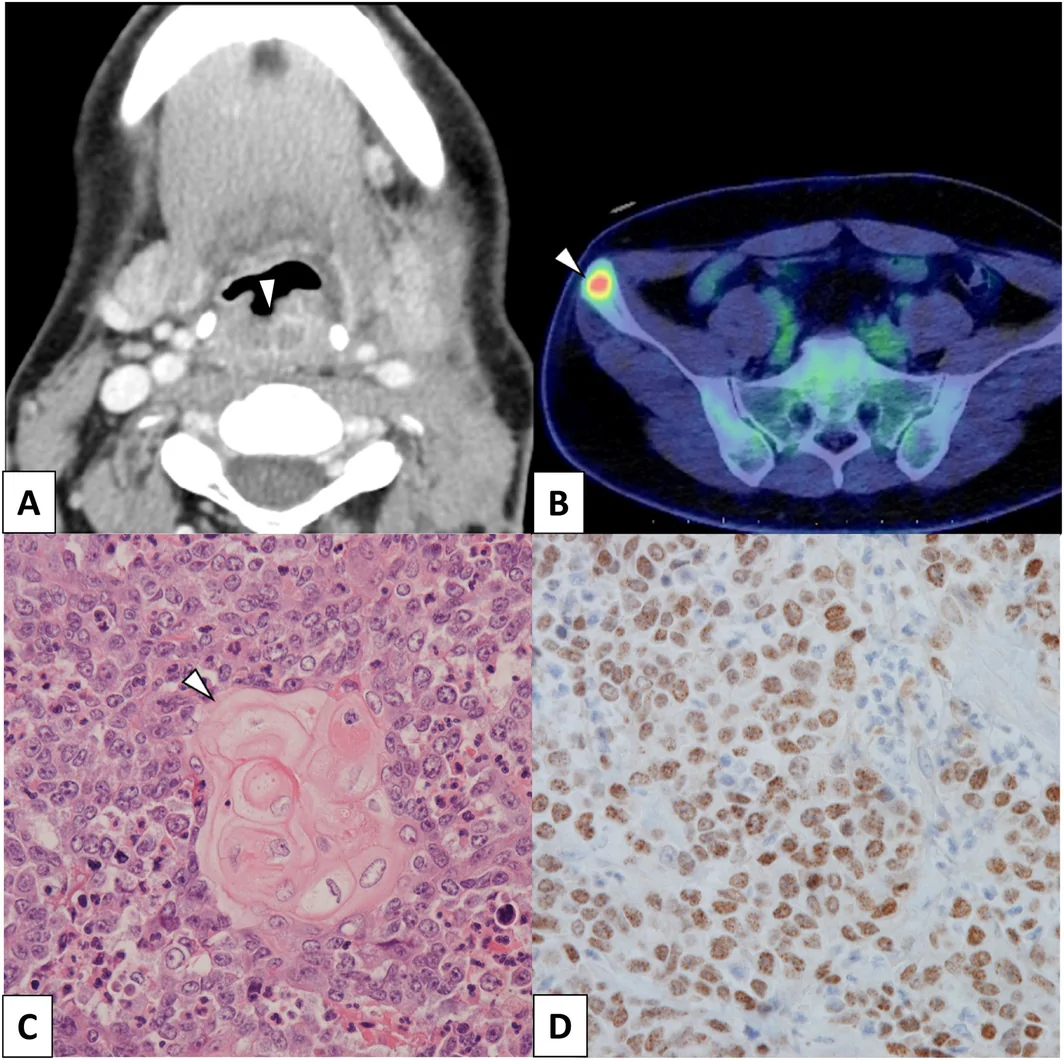
Recurrence at the posterior pharyngeal wall and definitive pathological diagnosis of NUT carcinoma. (A) Contrast‐enhanced CT image at the time of posterior pharyngeal wall recurrence, revealing an ulcerative tumor involving the middle‐to‐lower posterior pharyngeal wall (white arrow). (B) PET/CT image demonstrating fluorodeoxyglucose uptake in a solitary right iliac bone lesion (white arrow). (C) Histopathological image of the recurrent lesion (hematoxylin and eosin stain, original magnification ×400). Abrupt keratinization is observed (white arrow). (D) Immunohistochemical staining for NUT (original magnification ×400). Tumor cell nuclei show diffuse nuclear positivity with a characteristic speckled pattern. CT, computed tomography; PET, positron emission tomography; NUT, nuclear protein in testis.

In March 20X2, the patient developed odynophagia. Flexible nasolaryngoscopy and contrast‐enhanced CT revealed an ulcerative lesion involving the middle‐to‐lower posterior pharyngeal wall (Figure 3A), and biopsy confirmed SCC again. PET/CT demonstrated fluorodeoxyglucose uptake in the posterior pharyngeal wall and in a solitary iliac bone lesion, with no other metastatic lesions identified (Figure 3B).

Systemic therapy with cisplatin and docetaxel was initiated; however, imaging after the first cycle demonstrated further progression of the posterior pharyngeal lesion. The regimen was subsequently switched to nivolumab, but the tumor continued to enlarge, extending to the inferior margin of the nasopharynx and causing progressive narrowing of the posterior pharyngeal lumen. During this period, comprehensive genomic profiling of the recurrent tumor tissue was performed using FoundationOne CDx, a DNA‐based hybrid‐capture assay, which identified no actionable genomic alterations and did not detect the BRD4::NUTM1 fusion. Although the assay includes selected intronic coverage of NUTM1, rearrangement detection depends on breakpoint location, capture efficiency, and adequate supporting reads [23]; RNA‐based next‐generation sequencing was not performed. False‐negative results for NUTM1 rearrangements by next‐generation sequencing have also been reported in molecularly confirmed NUT carcinoma cases [24]. Therefore, the negative result did not exclude NUT carcinoma, and the diagnosis was subsequently established by NUT immunohistochemistry, as described below. At the time nivolumab was administered, the tumor had a programmed death‐ligand 1 combined positive score of 5, was microsatellite stable, and had a low tumor mutational burden of 4 mutations/Mb.

The posterior pharyngeal lesion progressed, whereas the iliac bone lesion remained stable without additional distant metastases. A multidisciplinary tumor board recommended aggressive salvage surgery.

### 2.4 Repeated Radical Salvage Surgeries and Diagnosis of NUT Carcinoma (20X2)

As reconstruction was anticipated to extend from the nasopharynx to the hypopharynx, the patient was referred to Aichi Cancer Center for definitive surgical management. In May 20X2, radical salvage surgery consisting of circumferential pharyngectomy and cervical esophagectomy was performed, followed by free jejunal reconstruction. Histopathological examination demonstrated extensive involvement of the posterior pharyngeal wall with invasion into the submucosal and muscular layers, and the diagnosis remained poorly differentiated SCC.

In October 20X2, 5 months after free jejunal reconstruction, a recurrent lesion developed around the permanent tracheostoma with invasion into the reconstructed jejunal segment. In November 20X2, resection of the peristomal region and the involved jejunal segment was performed, followed by redo jejunal reconstruction and revision of the permanent tracheostoma using a deltopectoral flap. Representative intraoperative findings are provided in Figure S1. Additional immunohistochemical analysis revealed diffuse nuclear positivity for NUT, with p63 positivity, partial p40 positivity, and a wild‐type p53 pattern, establishing the definitive diagnosis of NUT carcinoma (Figure 3C,D).

### 2.5 Further Recurrence and Treatment With a Bromodomain and Extra‐Terminal (BET) Inhibitor (January–August 20X3)

**FIGURE 4:**
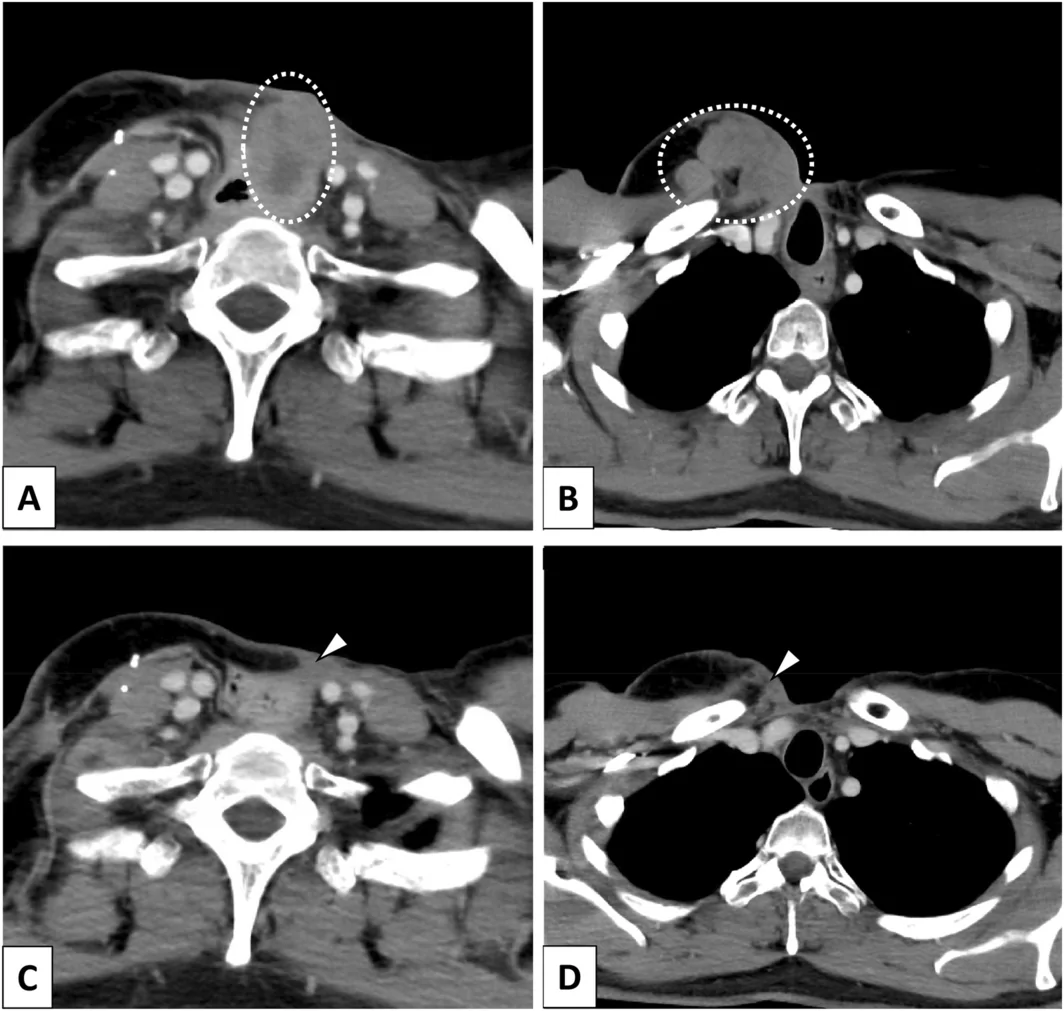
Therapeutic response to BET inhibitor therapy. Contrast‐enhanced CT images obtained in January 20X3 prior to initiation of BET inhibitor therapy. (A) Tumor infiltration into the wall of the free jejunal graft (white dotted circle). (B) Multiple metastatic lesions within and surrounding the deltopectoral flap (white dotted circles). Contrast‐enhanced CT images obtained 3 months after initiation of BET inhibitor therapy demonstrate marked tumor regression, consistent with a partial response. (C, D) Arrows indicate lesions corresponding to those shown in (A) and (B), respectively. BET, bromodomain and extra‐terminal; CT, computed tomography.

In January 20X3, recurrent disease was identified as tumor infiltration within the wall of the free jejunal graft and as multiple lesions involving both the intraluminal and surrounding tissues of the deltopectoral flap (Figure 4A,B). Following confirmation of the molecular diagnosis, treatment with a BET inhibitor was initiated as part of a clinical trial. The specific agent, dose, and schedule cannot be disclosed owing to trial confidentiality provisions; the agent was not discontinued for toxicity. The tumor showed a prompt clinical response, and imaging at 3 months demonstrated a marked partial response (Figure 4C,D). Nevertheless, after approximately 6 months of therapy, new lesions appeared, and treatment was discontinued after eight cycles due to disease progression.

Treatment with paclitaxel plus cetuximab was initiated; however, no objective clinical response was observed, and the disease continued to progress.

### 2.6 Alternating Vincristine, Doxorubicin, and Cyclophosphamide (VDC)/Ifosfamide and Etoposide (IE) and Subsequent Histone Deacetylase (HDAC)‐Based Epigenetic Intervention (November 20X3–April 20X4)

**FIGURE 5:**
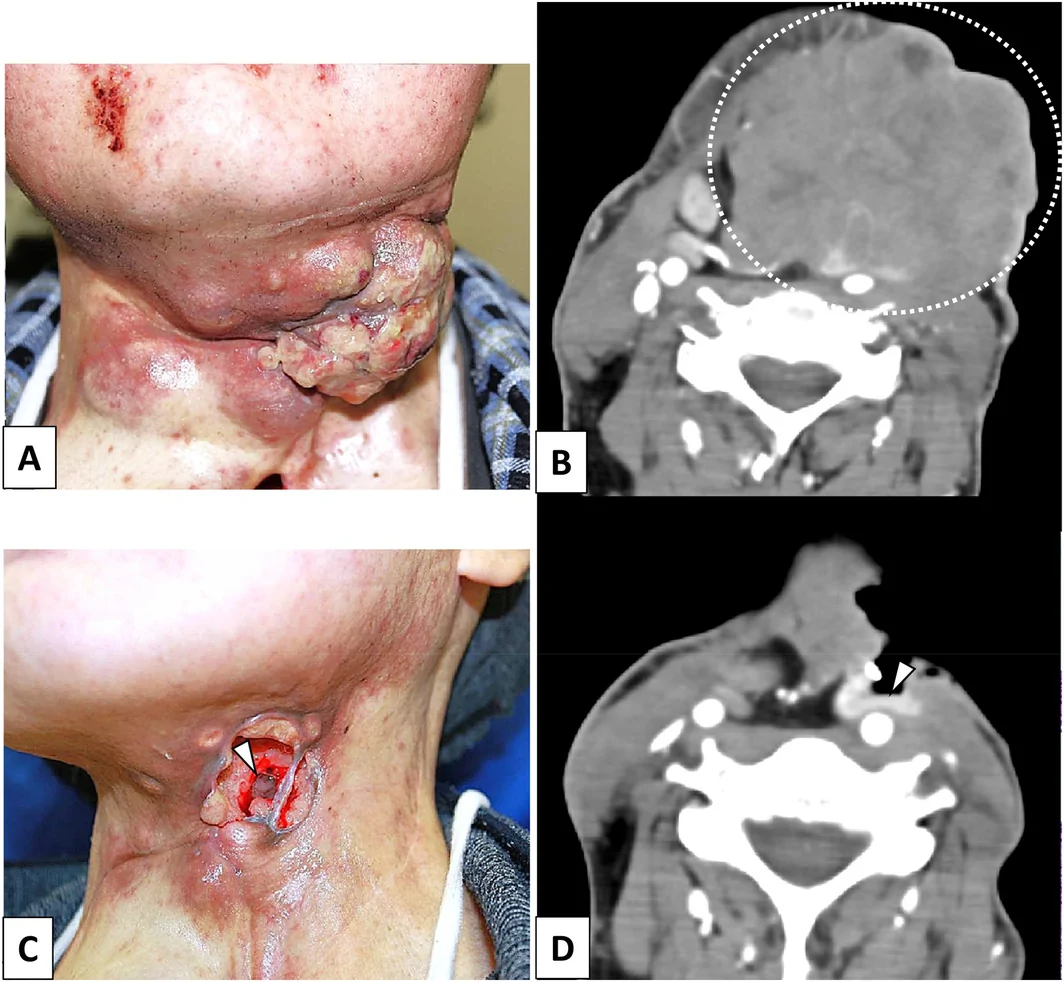
Therapeutic response to alternating VDC/IE chemotherapy. Before initiation of VDC/IE therapy. (A) Macroscopic appearance showing extensive cutaneous invasion and rapid tumor growth. (B) Contrast‐enhanced CT demonstrating a massive tumor infiltrating reconstructed tissues and lateral cervical structures (white dotted circle). After completion of two cycles of VDC/IE therapy: (C) Macroscopic view showing marked tumor shrinkage with formation of a large pharyngocutaneous fistula (white arrow). (D) Contrast‐enhanced CT confirming substantial tumor regression and the presence of the pharyngocutaneous fistula. The white arrow indicates the enhancing posterior wall of the remaining free jejunal graft. CT, computed tomography; IE, ifosfamide and etoposide; VDC, vincristine, doxorubicin, and cyclophosphamide.

By this stage, the disease demonstrated rapid progression with an increasingly infiltrative growth pattern involving both internal reconstructed tissues and external cervical structures (Figure 5A,B). Given the refractoriness to multiple prior systemic therapies, an intensive cytotoxic chemotherapy regimen was considered. Following institutional review board approval, alternating cycles of VDC and IE were administered. Notably, a marked partial response was achieved after two cycles, approximately 2 months after treatment initiation, as demonstrated by imaging obtained at the time of maximal tumor regression (Figure 5C,D). However, this control was not durable; during the third cycle, tumor regrowth became evident, and VDC/IE therapy was discontinued owing to disease progression. Subsequently, pembrolizumab combined with cisplatin and 5‐fluorouracil was administered for two cycles, but no objective clinical benefit was observed. Thereafter, the HDAC inhibitor vorinostat was initiated off‐label following expedited institutional approval. Vorinostat was administered orally at 400 mg once daily (four 100‐mg capsules) for 14 consecutive days. Despite the mechanistic rationale for targeting epigenetic dysregulation, no evidence of disease control was observed, and treatment was discontinued because of rapid tumor progression. No treatment‐related adverse events were observed. Finally, retreatment with paclitaxel plus cetuximab was attempted as a final salvage measure, but disease progression persisted.

### 2.7 Terminal Course and Palliative Management (June–July 20X4)

**FIGURE 6:**
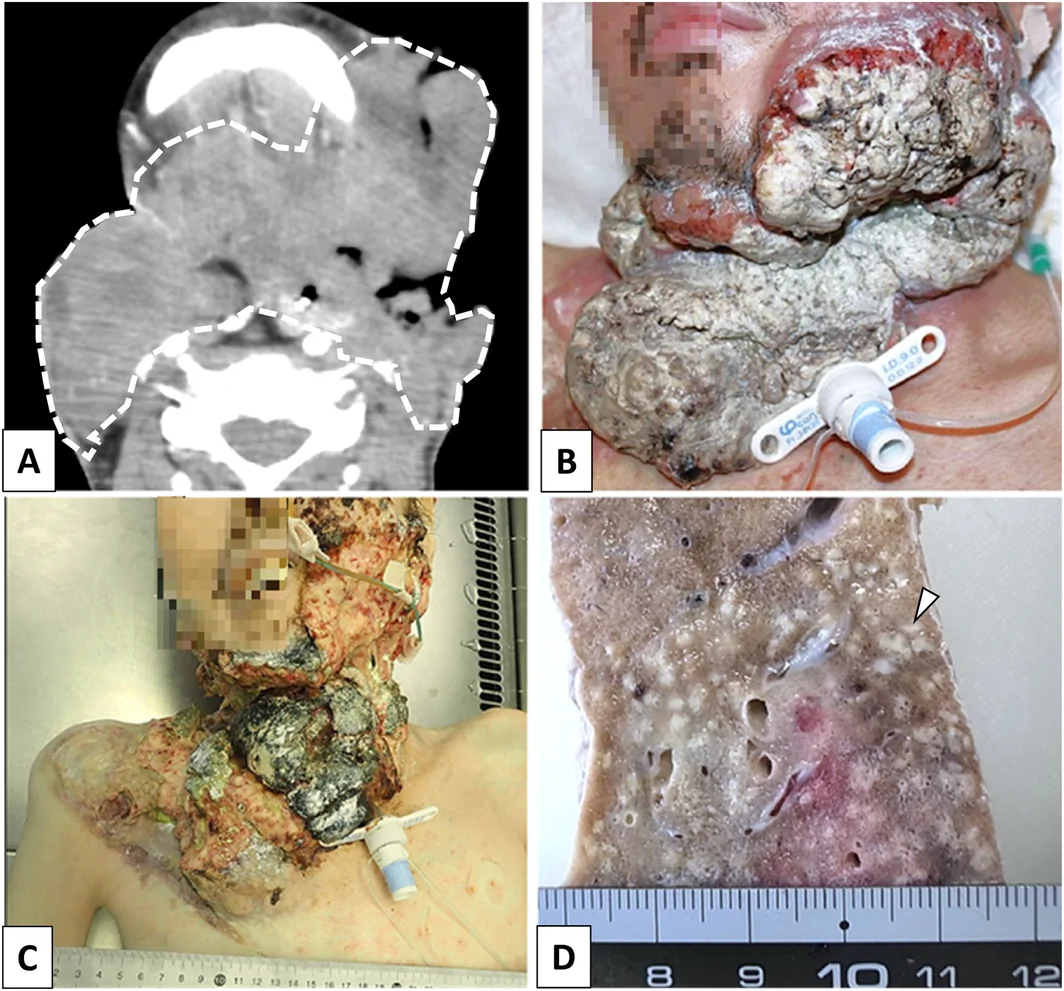
Terminal course and autopsy findings. (A) Contrast‐enhanced CT showing uncontrollable infiltrative tumor growth, with the anterior neck largely replaced by tumor tissue (white dotted area). (B) Macroscopic view following local treatment with Mohs paste, demonstrating effective control of exudate and bleeding owing to surface hardening. (C) Macroscopic finding during autopsy revealing a massive infiltrative tumor occupying the cervical region. (D) Macroscopic finding of the lung specimen at autopsy showing innumerable small nodular metastatic lesions (white arrows). CT, computed tomography.

In June 20X4, care was transitioned to palliative management. The tumor progressed in an uncontrollable and infiltrative manner (Figure 6A), accompanied by extensive necrosis with malodorous exudate and intermittent bleeding. Weekly application of Mohs paste was initiated to manage these symptoms and successfully limit further exophytic tumor growth at the treated surface (Figure 6B), as a palliative local measure [25, 26]. Pain and airway‐related discomfort were controlled with morphine. The clinical condition progressively deteriorated, and death occurred in late July 20X4 with his family present.

### 2.8 Autopsy Findings

A complete autopsy was performed with prior informed consent. At the time of death, the body mass index was 15.9 kg/m^2^ (weight, 43.4 kg), reflecting a profound weight loss of > 30 kg from baseline. A massive infiltrative tumor measuring 23 × 20 cm occupied the anterior neck and extended from the cheeks to the upper chest, with extensive cutaneous invasion, ulceration, and necrosis (Figure 6C). The reconstructed free jejunal graft was deeply infiltrated by the tumor.

In contrast to the severe locoregional disease, the distant metastatic burden was relatively limited. The lungs contained innumerable metastatic nodules, each only a few millimeters in diameter and not considered the primary cause of death (Figure 6D). Instead, the predominant pulmonary pathology consisted of bilateral lung abscesses with cavitary purulent destruction, primarily involving the lower lobes. Autopsy confirmed that the immediate cause of death was overwhelming infection secondary to uncontrolled locoregional tumor progression and airway compromise, rather than systemic metastatic disease.

## 3 Discussion

This case underscores the diagnostic challenges associated with laryngeal NUT carcinoma in routine clinical practice. In the present case, the tumor was histologically indistinguishable from poorly differentiated SCC; however, the patient's young age and non‐smoking status may have raised suspicion for a non‐conventional histologic entity.

The immunohistochemical findings, in particular the expression of squamous markers such as p63 and p40, further mimicked conventional SCC and contributed to the diagnostic pitfall. NUT carcinoma was not suspected until the recurrent tumor was evaluated at the specialized cancer center after an unexpectedly refractory clinical course. This diagnostic sequence underscores the importance of clinical suspicion, as routine histology and a limited immunohistochemical panel may fail to prompt NUT‐specific testing. Subsequent fluorescence in situ hybridization performed on archived formalin‐fixed specimens from the salvage total laryngectomy using a NUTM1 break‐apart probe confirmed that the tumor had been NUT carcinoma from the outset (Figure S2). Reverse transcription polymerase chain reaction (RT‐PCR) performed on archived formalin‐fixed, paraffin‐embedded biopsy specimens obtained at the time of the first local recurrence further identified a *BRD4*::*NUTM1* fusion, establishing the molecular diagnosis (Figure S3). Sanger sequencing of the RT‐PCR product confirmed the precise *BRD4*::*NUTM1* fusion junction, with the breakpoint sequence and corresponding transcript identifiers explicitly defined (Figure S4). Details of these molecular analyses are provided in Table S1, Supporting Information Methods.

Despite repeated salvage surgeries achieving gross and histologically complete resections, the disease recurred sequentially at non‐marginal, anatomically discontinuous sites, including the posterior pharyngeal wall, the peristomal region of the permanent tracheostoma, and ultimately within the reconstructed free jejunal conduit.

To contextualize this rare presentation, we compared the clinicopathological characteristics of the present case with those of the 18 previously reported cases of laryngeal NUT carcinoma summarized in a recent article (Table S2) [27]. Clinical outcomes were generally poor, with most patients dying of disease within the first year after diagnosis; durable survival was reported in only a small number of patients, some of whom harbored non‐BRD4 fusion partners. Most tumors arose in the supraglottic region, whereas only a small number were explicitly reported as originating from the vocal cord or glottis. The present case is notable for its glottic origin, an unusually prolonged clinical course of approximately 4 years, and, to our knowledge, the first autopsy‐based characterization of the terminal disease distribution in laryngeal NUT carcinoma.

Previous registry‐based analyses have demonstrated that head and neck NUT carcinoma with distant metastasis at presentation is associated with an extremely poor prognosis, with a reported 2‐year overall survival rate of 0% [6]. In contrast, despite the presence of iliac bone metastasis, our patient survived for > 2 years following its detection.

### 3.1 Resistance to Standard Head and Neck Squamous Cell Carcinoma (HNSCC) Therapies

In the present case, multiple systemic therapies conventionally used for HNSCC failed to achieve durable disease control. Induction chemotherapy with the TPEx regimen, subsequent platinum–taxane‐based chemotherapy, and immune checkpoint inhibitors, including nivolumab and pembrolizumab, all failed to suppress disease progression. In contrast, definitive radiotherapy with intensity‐modulated radiation therapy to a total dose of 70 Gy achieved temporary locoregional control, which was maintained for approximately 1 year. This clinical course is consistent with prior reports indicating that standard treatment strategies for HNSCC provide limited benefit in NUT carcinoma, with a median overall survival of approximately 6–9 months despite aggressive multimodal therapy [28]. These findings further support the concept that NUT carcinoma, despite its morphological resemblance to SCC, represents a biologically distinct disease entity that requires alternative therapeutic approaches [29].

However, the classification of NUT carcinoma as a distinct disease entity remains debated. Luo et al. recently proposed that NUT carcinoma arising in the lung or head and neck should instead be recognized as a molecularly defined subtype of SCC, based on shared transcriptional, histopathological, cell‐of‐origin, and molecular features [30]. Regardless of classification, this perspective further supports NUT‐specific testing in poorly differentiated SCC arising at these sites.

### 3.2 Experience With BET Inhibitor Therapy and HDAC Inhibition

The limited durability of response to BET inhibition is thought to reflect adaptive resistance mechanisms rather than the acquisition of target mutations [31, 32]. Tumor cells may rewire transcriptional regulatory networks and restore MYC‐centered transcriptional programs, thereby attenuating the therapeutic effect of BET inhibition [32, 33].

On this basis, HDAC inhibition has been explored as a complementary strategy, as these enzymes regulate the same acetylation‐dependent transcriptional circuitry from a different regulatory axis [33]. Although preclinical studies have demonstrated synergistic antitumor effects with combined BET and HDAC inhibition [32, 34], clinical translation has been limited by overlapping toxicities and modest efficacy in NUT carcinoma [32, 35, 36]. In the present case, single‐agent vorinostat failed to achieve meaningful disease control. The limited activity of BET and HDAC inhibitors may involve distinct resistance mechanisms. Proposed mechanisms include adaptive maintenance or reactivation of oncogenic transcription following BET inhibition [31, 32, 33] and functional redundancy, particularly between HDAC1 and HDAC2, following HDAC inhibition [36]. However, these mechanisms were not investigated in the present case.

### 3.3 Transient Tumor Control Achieved With VDC/IE Therapy

In NUT carcinoma, sporadic responses to chemotherapy regimens typically used for non‐squamous malignancies have been reported. Based on these observations, alternating cycles of VDC and IE were administered in the present case, resulting in a partial response that was maintained for approximately 4 months.

VDC/IE is an intensive cytotoxic regimen that induces DNA damage and disrupts cell‐cycle progression, rather than directly targeting the transcriptional dysregulation driven by BRD4::NUTM1 fusion proteins. The transient response observed in this case indicates that a highly proliferative NUT carcinoma may retain limited sensitivity to cytotoxic stress, although durable disease control with chemotherapy alone remains uncommon.

Consistent with prior reports, long‐term survival in patients with NUT carcinoma has generally been achieved through multimodal strategies emphasizing durable locoregional control with surgery and radiotherapy, in combination with systemic therapy [37]. In the present case, prior high‐dose radiotherapy precluded further integration of radiotherapy with VDC/IE therapy.

The autopsy findings indicate that fatal outcomes in NUT carcinoma may be driven predominantly by relentless locoregional progression along the aerodigestive tract rather than by systemic metastatic burden, consistent with prior reports emphasizing the prognostic importance of durable locoregional control [37]. The immediate cause of death was overwhelming infection secondary to uncontrolled locoregional disease at the permanent tracheostoma.

### 3.4 Implications for Therapeutic Development Suggested by the Present Case

The uniformly transient nature of treatment effects in this case suggests that NUT carcinoma is sustained by a transcription‐dependent network that is unlikely to be durably disrupted by inhibition of a single node. In solid tumors, BET inhibition primarily induces cell‐cycle arrest and terminal differentiation rather than direct cytotoxicity. Therapeutic escape is frequently mediated by the compensatory recruitment of transcriptional co‐regulators, such as p300/CBP or HDACs, and the activation of downstream bypass signaling pathways, including the MAPK/ERK cascade [31, 32, 33]. Accordingly, BET inhibition should be considered within a multimodal, phase‐adapted strategy that integrates rational combinations targeting complementary pathways with cytotoxic debulking and definitive locoregional control through surgery and radiotherapy. Improving outcomes will likely require treatment sequencing aligned with the underlying oncogenic driver mechanisms rather than the simple addition of successive agents.

Treatment selection and initiation were determined through multidisciplinary tumor board discussions. Decisions to discontinue each therapy were made by the treating physician on an individualized basis, primarily considering radiographic or clinical disease progression and the absence of an objective response rather than according to a fixed institutional protocol.

As a single case report, these observations cannot be generalized to all patients with NUT carcinoma, and the sequential administration of therapies precludes direct comparison of treatment efficacy. Nevertheless, the present clinical course provides mechanistic insights into therapeutic resistance and highlights the importance of biologically informed multimodal strategies in this disease.

Early recognition through targeted molecular testing and timely consideration of investigational or molecularly guided treatment strategies may be critical to improving outcomes. Continued accumulation of detailed clinical and pathological data, particularly from cases with long‐term longitudinal follow‐up, will be essential for advancing the understanding and management of this rare disease.

## Author Contributions

**Masafumi Kanno:** conceptualization, methodology, investigation, validation, writing – review and editing, writing – original draft, visualization, funding acquisition. **Chiai Sasaki:** investigation, conceptualization, methodology, visualization. **Eiichi Kato:** formal analysis, validation, methodology, writing – review and editing. **Hoshino Terada:** methodology, investigation, visualization, writing – review and editing. **Nobuhiro Hanai:** methodology, visualization, writing – review and editing, investigation. **Eiichi Sasaki:** investigation, methodology, visualization, writing – review and editing. **Mana Fukushima:** investigation, methodology, visualization, writing – review and editing. **Toshiki Masuishi:** investigation, methodology, visualization, writing – review and editing. **Keisuke Takanari:** writing – review and editing, visualization, methodology, investigation. **Yasuto Fukada:** investigation, conceptualization, methodology. **Yuto Miyazaki:** methodology, conceptualization, investigation. **Yuki Sonoda:** investigation, conceptualization, methodology. **Yukinori Kato:** methodology, conceptualization, investigation, writing – review and editing. **Taiyo Morikawa:** conceptualization, investigation, methodology, writing – review and editing. **Tetsuji Takabayashi:** writing – review and editing, supervision, project administration. **Shigeharu Fujieda:** project administration, supervision, writing – review and editing.

## Funding

This work was supported by the Japan Society for the Promotion of Science (JSPS) KAKENHI (grant number 25K12740).

## Ethics Statement

This study was conducted in accordance with the Declaration of Helsinki. The publication of this case report was approved by the Institutional Review Board of the University of Fukui on January 29, 2026 (approval number: 20250230). All identifiable information was anonymized.

## Consent

Written informed consent for publication was obtained from the patient's family and documented in the electronic medical record.

## Conflicts of Interest

Masafumi Kanno reports that GE Healthcare provides PET/MRI analysis software to his institution for clinical use. Toshiki Masuishi reports research grants to his institution from MSD, Daiichi Sankyo, Ono, Novartis, Amgen, Boehringer‐Ingelheim, Pfizer, Eli Lilly, AbbVie, Merck Biopharma, and others; advisory role for Nippon Kayaku; and lecture honoraria from multiple pharmaceutical companies, outside the submitted work. The other authors declare no conflicts of interest.

## Supporting information


**Table S1:** Molecular analyses for identification of the *BRD4::NUTM1* fusion primers used for RT‐PCR.
**Table S2:** Previously reported cases of laryngeal NUT carcinoma compared with the present case.

**Figure S1:** Intraoperative findings during salvage surgeries.
**Figure S2:** Fluorescence in situ hybridization analysis of *NUTM1* gene rearrangement.
**Figure S3:** RT‐PCR analysis of the *BRD4::NUTM1* fusion gene.
**Figure S4:** Sanger sequencing of the fusion junction.

## Data Availability

The data supporting the findings of this study are available within the article and its Supporting Information Materials. No additional datasets were generated or analyzed.
