# Supplementary material for: Laryngeal NUT Carcinoma With BRD4 :: NUTM1 Fusion: A 4‐Year Clinical Course Highlighting the Limitations of Current Multimodal Therapy
Source: Cancer Rep (Hoboken). 2026 Sep 8;9(9):e70679. doi: 10.1002/cnr2.70679 (PMC13553551; doi:10.1002/cnr2.70679)
Supplement: Supplementary file 1 [file CNR2-9-e70679-s002.docx]

**Supplementary Table S1. Molecular analyses for identification of the *BRD4::NUTM1* fusion**

**Primers used for RT-PCR**

| **Target gene** | **Primer name** | **Sequence (5'–3')** | **Orientation** |
| --- | --- | --- | --- |
| BRD4 | BR2276F | AAGTTGATGTGATTGCCGGCTCCTC | Forward |
| NUT | NUT294R | CTGAAGGCATGATGGGCTGTGG | Reverse |

Expected amplicon size: approximately 255 bp

RT-PCR, reverse transcription polymerase chain reaction; NUT, nuclear protein in testis

**Supplementary Table S2. Previously reported cases of laryngeal NUT carcinoma compared with the present case.**

| **No.** | **Author (Year)** | **Age/Sex** | **Subsite** | **Fusion / diagnostic basis** | **Outcome** |
| --- | --- | --- | --- | --- | --- |
| 1 | Vargas et al. (2001) | 13/F | Epiglottis | FISH: BRD4::NUT | Died (9 mo) |
| 2 | Stelow et al. (2008) | 78/F | Supraglottis | FISH: BRD4::NUT | Died (8 mo) |
| 3 | Hellquist et al., Toronto (2017) | 5/F | Larynx / base of tongue | IHC: NUT+ | Died (7 mo) |
| 4 | Hellquist et al., Chicago (2017) | 41/M | Supraglottis | FISH+IHC: BRD4::NUT | Died (11 mo) |
| 5 | Hellquist et al., Porto (2017) | 47/M | Larynx / base of tongue | IHC: NUT+ | Died (3 mo) |
| 6 | Hellquist et al., Auckland (2017) | 17/M | Supraglottis | FISH+IHC: BRD4::NUT | Alive with disease |
| 7 | Kundra et al. (2016) | 39/M | Supraglottis | IHC: NUT+ | N/A |
| 8 | Vorstenbosch et al. (2018) | 13/M | Epiglottis | Genetics: BRD3::NUT | Remission (>5 y) |
| 9 | Zhang et al. (2020) | 20/F | Left vocal cord | IHC: NUT+ | Remission (>26 mo) |
| 10 | Zhou et al. (2020) | 35/F | Larynx | IHC+FISH: NUT+ | Died (9 mo) |
| 11 | Higashino et al. (2022) | 22/F | Supraglottis (arytenoid) | IHC: NUT+ | Died (8 mo) |
| 12–13 | Viswanathan et al. (2024) | Cohort | Larynx (2 cases) | IHC: NUT+ (72% BRD4::NUT in cohort) | 3-y DSS 38% (cohort) |
| 14 | Ugurluer et al. (2024) | 17/M | Epiglottis | FISH: NUTM1 rearrangement | Died (15 mo) |
| 15 | Liu et al., Case 7 (2025) | 72/F | Arytenoid / AE fold | Genetics+FISH: BRD4::NUT | Died (2 mo) |
| 16 | Liu et al., Case 14 (2025) | 35/M | Vocal cord / subglottis | Genetics: NSD3::NUT | Alive (3 mo) |
| 17 | Liu et al., Case 15 (2025) | 52/M | Epiglottis / arytenoid | FISH: NUTM1 rearrangement | Alive (3 mo) |
| 18 | Arora et al. (2025) | 27/M | Supraglottis / ant. commissure | IHC: NUT+ (aberrant LCA+) | Died (~4 mo) |
| 19 | Present case | 30/M | Glottis / supraglottis | IHC+FISH+RT-PCR+Sanger: BRD4::NUTM1 | Died (~4 y); autopsy-confirmed |

AE, aryepiglottic; ant., anterior; DSS, disease-specific survival; FISH, fluorescence in situ hybridization; IHC, immunohistochemistry; LCA, leukocyte common antigen; N/A, not available; RT-PCR, reverse transcription polymerase chain reaction. Cases 1–18 were summarized from the recent article by Coca-Pelaz and Ferlito (2026); the present case was added for comparison.

**Supplementary Methods. RNA extraction**
Total RNA was extracted according to the manufacturer’s recommended protocol using the RNeasy FFPE Kit (QIAGEN).

**Reverse transcription (RT)**
Reverse transcription was performed in a total reaction volume of 20 μL using the High-Capacity complementary DNA (cDNA) Reverse Transcription Kit (Applied Biosystems), containing the following: 10× RT Buffer, 25× deoxynucleotide triphosphate (dNTP) Mix (100 mM), 10× RT Random Primers, MultiScribe Reverse Transcriptase (50 U/μL), RNase inhibitor, nuclease-free H₂O, and RNA samples.
Incubation conditions were 37°C for 120 min, followed by enzyme inactivation at 85°C for 5 min, and holding at 4°C.

**Polymerase chain reaction (PCR)**
PCR amplification was performed using a hot-start Taq DNA polymerase (Quick Taq HS DyeMix; TOYOBO) at 1 U per reaction in a total volume of 50 μL containing nuclease-free H₂O, 2× Quick Taq HS DyeMix, forward primer (BR2276F), reverse primer (NUT294R), and 1 μL of cDNA template.

**PCR cycling conditions**
Initial denaturation at 94°C for 2 min, followed by 45 cycles of denaturation at 94°C for 30 s, annealing at 60°C for 30 s, and extension at 68°C for 30 s, with a final extension at 72°C for 5 min.

**Gel electrophoresis and purification**
PCR products were resolved on a 2.0% agarose gel and visualized using GelRed nucleic acid gel stain (Biotium). Bands corresponding to the expected amplicon size were excised and purified using a commercial gel extraction kit (Gel/PCR Extraction Kit; FastGene).

**Sanger sequencing**
Purified PCR products were subjected to Sanger sequencing using dye-terminator chemistry (BigDye Terminator v3.1; Thermo Fisher Scientific). Sequencing was performed using the same primers as for RT-PCR. Reverse reads were converted to the 5' *BRD4*–3' *NUTM1* orientation prior to alignment with reference transcripts (BRD4: NM_001379291.1; *NUTM1*: NM_001284292.2). Sequence analysis confirmed an in-frame *BRD4::NUTM1* fusion junction.

**Supplementary Figure Legends**

**Supplementary Figure S1. Intraoperative findings during salvage surgeries.**

**Initial pharyngolaryngoesophagectomy with free jejunal reconstruction:**
(A) Intraoperative view after circumferential pharyngectomy and cervical esophagectomy, achieving a surgical margin > 2 cm.
(B) Free jejunal reconstruction extending to the nasopharynx.
**Revision of free jejunal reconstruction:**
(C) Complete resection of the recurrent lesion, including involved skin, permanent tracheostoma, and a portion of the previous free jejunal graft.
(D) Redo free jejunal reconstruction and revision of the permanent tracheostoma using a deltopectoral flap.

**Supplementary Figure S2. Fluorescence *in situ* hybridization analysis of *NUTM1* gene rearrangement.**

Fluorescence *in situ* hybridization analysis was performed using a formalin-fixed, paraffin-embedded specimen obtained at the time of the initial total laryngectomy. *NUTM1* gene rearrangement is observed in tumor cell nuclei (white arrows).

**Supplementary Figure S3. RT-PCR analysis of the *BRD4::NUTM1* fusion gene.**

RT-PCR analysis demonstrating the presence of a *BRD4::NUTM1* fusion transcript.

RT-PCR, reverse transcription polymerase chain reaction

**Supplementary Figure S4. Sanger sequencing of the fusion junction.**

Sanger sequencing results of the RT-PCR product are shown. Chromatogram analysis clearly identifies the fusion junction between exon 11 of the *BRD4* gene and exon 3 of the *NUTM1* gene. Identification of the breakpoint sequence confirms the fusion structure at the transcript level.

RT-PCR, reverse transcription polymerase chain reaction
